# A systematic review of [^68^Ga]Ga-DOTA-FAPI-04 and [^18^F]FDG PET/CT in the diagnostic value of malignant tumor bone metastasis

**DOI:** 10.3389/fonc.2022.978506

**Published:** 2022-11-10

**Authors:** Lanying Li, Xin Hu, Jiao Ma, Songsong Yang, Weidong Gong, Chunyin Zhang

**Affiliations:** ^1^ Department of Nuclear Medicine, The Affiliated Hospital of Southwest Medical University, Luzhou, China; ^2^ The Second People’s Hospital of Yibin, Yibin, China; ^3^ Department of Nuclear Medicine, The No.2 People’s Hospital of Yibin, Yibin, China; ^4^ Nuclear Medicine and Molecular Imaging Key Laboratory of Sichuan Province, Luzhou, China; ^5^ Academician (Expert) Workstation of Sichuan Province, Luzhou, China

**Keywords:** [^68^Ga]Ga-DOTA-FAPI-04, [^18^F]FDG PET/CT, malignant tumor bone metastasis, systematic review, cancer

## Abstract

**Objective:**

This study aims to perform a systemic analysis of [^68^Ga]Ga-DOTA-FAPI-04 positron emission tomography (PET)/computerized tomography (CT) and [^18^F]FDG PET/CT for the diagnosis of malignant tumor bone metastasis based on existing clinical evidence.

**Methods:**

This systematic review followed the guidelines of the Preferred Reporting Project (PRISMA) for systematic reviews and meta-analysis. This is a retrospective study of articles published in PubMed. Embase was searched online from the start of May 2022. The main endpoints were the maximum standardized uptake value and the tumor-to-background ratio to determine the examination performance of [68Ga]Ga-DOTA-FAPI-04 and [^18^F]FDG for bone transfer stoves. Based on the entry and discharge standards, two researchers extracted documents and data and then performed the quality evaluation.

**Results:**

A total of eight studies on the metastasis of malignant tumors on bone were included, which involved 358 patients in the final analysis.

**Conclusion:**

[^68^Ga]Ga-DOTA-FAPI-04 showed better detection performance for bone metastasis. The sensitivity of [^68^Ga]Ga-DOTA-FAPI-04 for the diagnosis of the primary tumor was higher than that of [^18^F]FDG, whereas the specificity of [^18^F]FDG was higher than that of [^68^Ga]Ga-DOTA-FAPI-04. However, further randomized controlled trials and prospective clinical trials are warranted to compare the diagnostic performance of [^68^Ga]Ga-DOTA-FAPI-04 PET/CT and [^18^F]FDG PET/CT.

**Systematic review registration:**

https://www.crd.york.ac.uk/PROSPERO/, identifier (CRD42022313019)

## Introduction

Malignant bone metastasis usually leads to the development of a series of complications, such as acute pain, pathogenic fractures, and spinal disease, which are related to bone metastasis. Bone metastasis directly affects the treatment strategy and leads to a poor prognosis (1–4). Treatment methods for bone metastasis include systemic therapy (hormones, analgesics, chemotherapy, bisphosphonates, etc.), external beam radiotherapy, surgery, and radionuclide-targeted therapy (5–8). Studies on bone metastasis biology have led to the development of drugs for bone metastasis treatment: zoledronate (Zol) and denosumab (Den). In recent years, many scholars continue to study bone metastasis biology. These studies are about colony-stimulating factor-1 (CSF-1), epidermal growth factor receptor (EGFR), Jagged1, etc. These studies indicate broad potential applications for bone metastasis prevention or treatment (9–11). Therefore, discovering this metastasis to make accurate disease-staging and developing guidance management strategies are essential for improving comfort and the survival rate of the patients (12).

The present imaging methods commonly used for tumor bone metastasis diagnosis and installment include [^99m^Tc]Tc-MDP and [^18^F]FDG positron emission tomography (PET)/computerized tomography (CT). [^99m^Tc]Tc-MDP is used to conduct conventional bone scanning for the entire body. The [^99m^Tc]Tc-MDP flashing scan depends on the identification of bone reactions of the accumulated bones instead of detecting the tumor itself. Although the technology is sensitive to the detection of advanced bone metastases, early diagnosis can be overlooked (13). At present, [^18^F]FDG PET/CT has been recognized as an effective imaging method for detecting tumor appearance (14).

However, owing to the stimulation of hematopoietic cytokines or the presence of benign lesions (such as fractures, osteoma, fibroblast hyperplasia, eosinophils, cartilage mucus fibroma, fibrous hyperplasia fibers tumor, thyroid glandular osteopathy, aneurysmal osteoma, and non-ossified fibroma), an abnormal intake of [^18^F]FDG radioactive tracer in the bone can occur, which limits the ability of this method to a certain extent to detect bone metastasis (15). Moreover, [^18^F]FDG intake can be weak, especially in different tissue subtypes, which significantly reduces the sensitivity of diagnosis. Therefore, developing an effective PET tracer is expected to improve the treatment strategy of patients with tumor bone metastasis as well as ensure the individual care of the patients.

Cancer-associated fibroblast (CAF) is an important part of the microenvironment of a tumor that plays an important role in tumor growth, immunosuppression, and tumor invasion (16). Fiber cell activation protein (FAP) is a type II membrane-binding glycoprotein belonging to dipeptidyl peptidase 4. It is expressed in cancer-related fibroblasts and is the main component of epithelial tumors (17). Some researchers have proposed the hypothesis that FAP activity affects the growth, invasion, and metastasis of a tumor (18). Therefore, theoretically, FAP-targeted imaging can be considered a promising strategy for the visualization of various tumors and non-tumor diseases. In this study, we aimed to determine the accuracy of [^68^Ga]Ga-DOTA-FAPI-04 PET/CT and [^18^F]FDG PET/CT in assessing the diagnostic value of tumor bone metastasis.

## Materials and methods

This systematic review was conducted in accordance with the Preferred Reporting Items for Systematic Reviews and Meta-Analysis (PRISMA) guidelines (19).

### Search strategy

The articles on [^68^Ga]Ga-DOTA-FAPI-04 PET/CT and [^18^F]FDG PET/CT in malignant tumor bone metastasis were searched across PubMed and Embase for those published until May 2022. The search keywords used included [FAPI] AND [FDG]. We searched all research papers and incorporated the appropriate data for analysis. If the articles met the research standards, the full text was retrieved. In the case of a duplicate (data from the same test or institution), only the most complete, latest, and related studies were selected. No limit was placed on the type of research.

### Inclusion criteria

① Research object: No fewer than three people who had been diagnosed with pathological diagnosis or imaging follow-up, including medical imaging, physical examination, and laboratory examination outcomes, who were then confirmed as patients with malignant tumor bone metastases, including new diagnosis and tumor recurrence or progress. Patients with recurrence and metastases of the tumor with an interval between treatment and undertaking PET/CT for >6 months. If the data were from the same research group, these were included in the study of the highest number of patients.

② Intervention: [^68^Ga]Ga-DOTA-FAPI-04 PET/CT and [^18^F]FDG PET/CT were used for appearance, and the interval between the appearance was not more than 2 weeks.

③ Ending: The average SUVmax and TBR of [^68^Ga]Ga-DOTA-FAPI-04 PET/CT and [^18^F]FDG PET/CT were obtained. The sensitivity and specificity of patients with [^68^Ga]Ga-DOTA-FAPI-04 PET/CT and [^18^F]FDG PET/CT for new diagnosis and tumor recurrence or progress were recorded.

No limit was set for the type of research.④

### Exclusion standards

① Patients who undertook other treatments such as chemotherapy and radiotherapy during imaging examination, accompanied by severe low leukocytes, low platelets, and liver or renal failure.

② Bone benign lesions, including benign tumors, fractures, and bone infections.

③ Malignant tumor, originally in the bone.

④ Repeatedly published research, meta-analysis, review, case reports, brief communication, abstracts, and letters to the editors were excluded.

⑤ No voluntary or signed consent from the patient.

### Quality assessment

**Figure 1:**
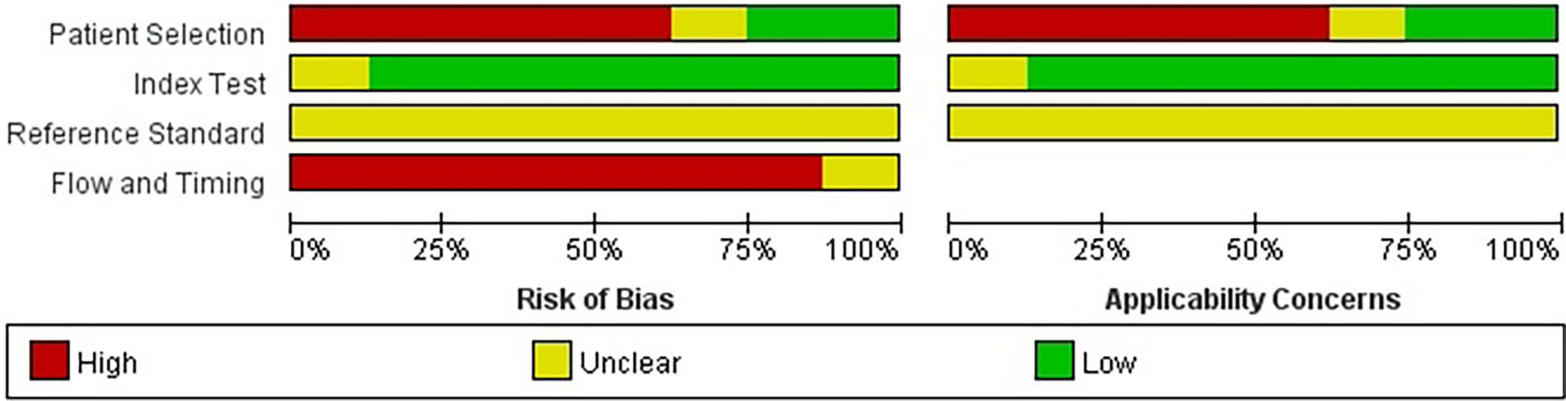
The quality assessment results of the studies.

**Figure 2:**
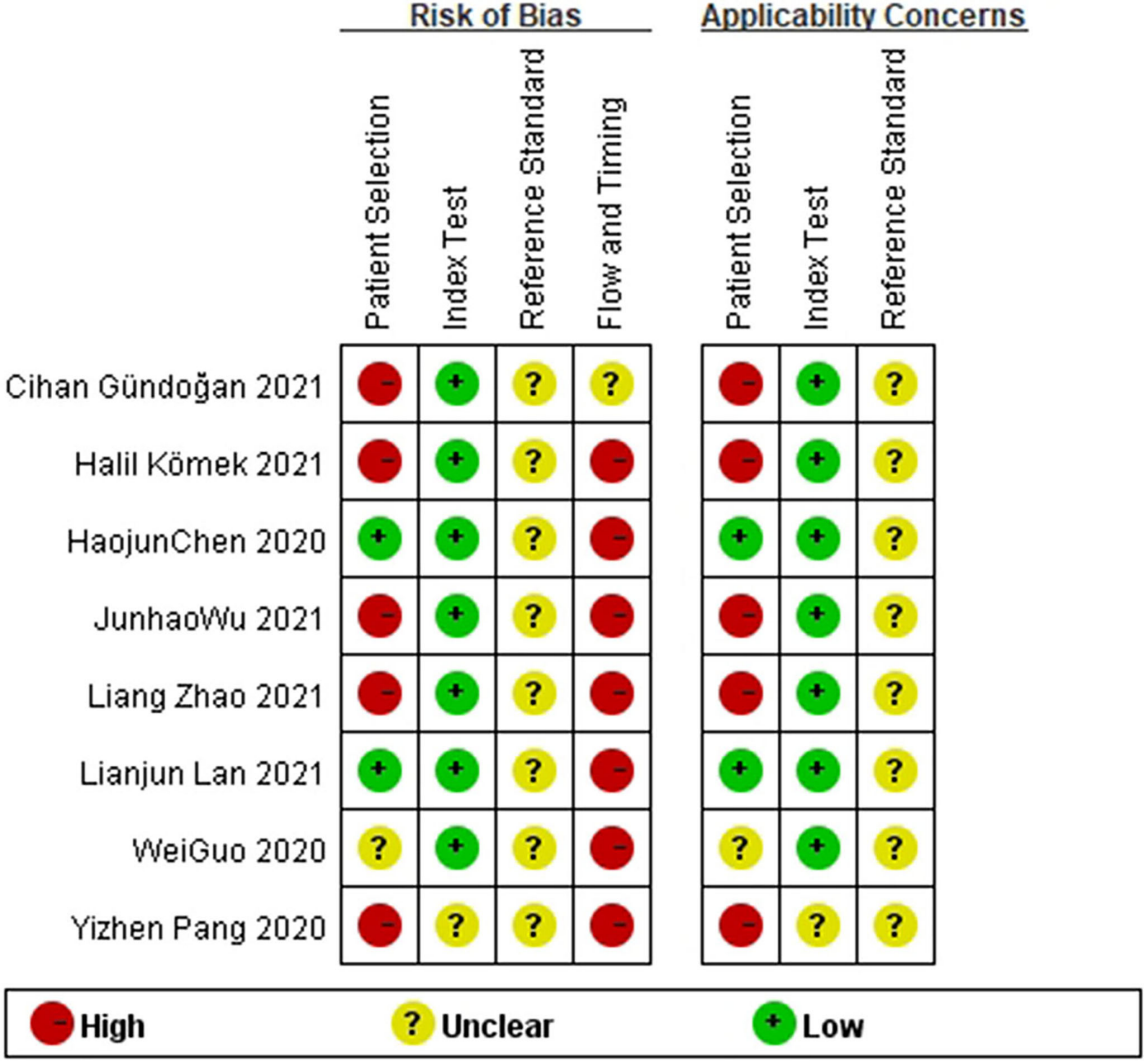
The quality assessment results of the studies.

Two researchers independently conducted literature retrieval and extraction of the assessment materials. In the case of a difference in opinions, the matter was discussed with third parties. A total of eight studies were screened, and the QUADAS-2 meter was used for the quality evaluation of the research (**Figures 1**, **2**).

## Results

### Literature retrieval results

**Figure 3:**
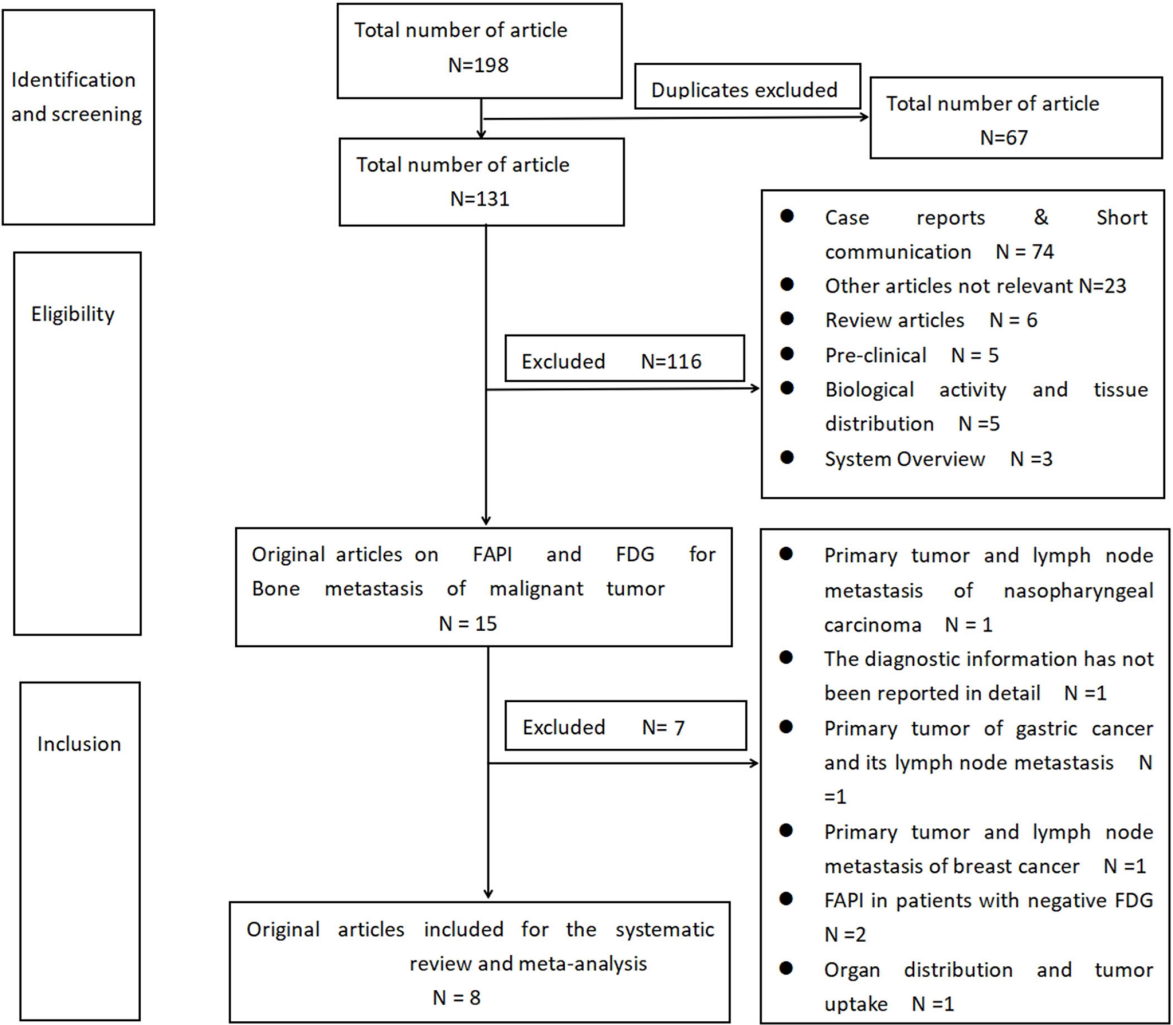
Flowchart of literature screening.

Based on the specified search strategy, a total of 198 related articles were extracted, of which 67 repeated articles were excluded. By reading the title and summary, 116 articles were excluded, which included 74 cases and brief exchanges, 23 articles that were not related to the topic, six reviews, five clinical studies, five articles on the biological activity and tissue distribution of [^68^Ga]Ga-DOTA-FAPI-04 in the body, and three meta-analyses. By reading the complete text, seven articles were excluded based on the integration and exclusion standards of this study. Past studies by Chen et al. and Gu et al. only detected the diagnostic value of [^68^Ga]Ga-DOTA-FAPI-04 among the FDG-negative patients, and the risk of bias was greater; therefore, these studies were excluded (20, 21). Qin et al. determined the diagnostic value of [^68^Ga]Ga-DOTA-FAPI-04 in primary tumors and lymph node metastasis of nasopharyngeal carcinoma (22). Dendl et al. studied the biological distribution and tumor intake of [^68^Ga]Ga-DOTA-FAPI-04 in gynecological malignant tumors (23). Jiang et al. studied the diagnostic performance of primary lesions and the lymph node metastasis of [^68^Ga]Ga-DOTA-FAPI-04 in gastric cancer (24). An article by Elboga et al. did not report the information on the diagnosis of the two imaging agents in the bone metastatic tumor (25). An article by Giesel et al. studied the organ distribution and tumor intake of the two imaging agents in different cancer patients (26). Finally, a total of eight articles were included, as shown in **Figure 3** **(** 12, 14, 27–32). The basic characteristics of the selected research object and the observation indicator of the research object are shown in **Tables 1**–**3**.

**Table 1:** Basic characteristics of incorporated research object.

Article	Primary tumor	Man (*n*)	Woman (*n*)	Patients (*n*)	Age (median, range)	Scan interval time (day)
Zhao et al. (2021) (27)	Nasopharyngeal carcinoma	35	10	45	50 (25–70)	2 (1–14)
Gündoğan et al. (2022) (31)	Gastric adenocarcinoma	12	9	21	61 (40–81)	<1 week
Kömek et al. (2021) (30)	Breast cancer	0	20	20	44 (32–65)	<1 week
Chen et al. (2020) (32)	Various types	47	28	75	61.5 (32–85)	<1 week
Wu et al. (2021) (12)	Lung cancer, thyroid cancer, and liver cancer	18	12	30	58 (25–78)	<1 week
Guo et al. (2020) (14)	Liver cancer	34	25	9	60.6 (33–75)	<1 week
Pang et al. (2020) (28)	Gastric, duodenal, and colorectal cancers	18	17	35	64 (53–68)	2 (1–6)
Lan et al. (2022) (29)	Various types	69	54	123	56.11 ± 11.94	3

**Table 2:** Patients of bone metastases detected by [^68^Ga]Ga-DOTA-FAPI-04 and [^18^F]FDG PET/CT and the sensitivity and specificity.

Article	Tumor stage	[^68^Ga]Ga-DOTA-FAPI-04		[^18^F]FDG	
		Sensitivity (%)	Specificity (%)	Sensitivity (%)	Specificity (%)
Zhao et al. (2021) (27)	Primary = 39	100%	NR	97%	NR
	Recurrence = 6	100%	33.4%	66.7%	100%
Gündoğan et al. (2022) (31)	Primary = 15	100%	100%	86.6%	100%
	Recurrence = 6	100%	83.3%	100%	100%
Kömek et al. (2021) (30)	Primary = 15	100%	95.6%	78.2%	100%
	Recurrence = 5	NR	NR	NR	NR
Chen et al. (2020) (32)	Primary = 54	98.2%	NR	82.1%	NR
	Recurrence = 21	100%	100%	57.1%	100%
Wu et al. (2021) (12)	Primary = 30	100%	NR	95.7%	NR
	Recurrence = 0	NR	NR	NR	NR
Guo et al. (2020) (14)	Primary = 34	96%	NR	65%	NR
	Recurrence = 0	NR	NR	NR	NR
Pang et al. (2020) (28)	Primary = 19	100%	NR	53%	NR
	Recurrence = 16	100%	67%	44%	94%
Lan et al. (2022) (29)	Primary = 82	97.67%	NR	84.89%	NR
	Recurrence = 20	NR	NR	NR	NR

NR, not reported.

**Table 3:** Comparison of [^68^Ga]Ga-DOTA-FAPI-04 and [^18^F]FDG uptake in bone metastases.

Article	Patients (*n*)	No. of total lesions	SUVmax (median, range)	No. of positive lesions	SUVmax (median, range)	No. of positive lesions	*p* (SUVmax)	TBR (median, range)	TBR (median, range)	*p* (TBR)
Zhao et al. (2021) (27)	5	19	9.11 (3.01–20.41)	19	1.86 (0.66–11.55)	7	<0.001			
Gündoğan et al. (2022) (31)	4	NR	4.6 (1.2–10.7)	117	9.0 (1.6–21.1)	101	<0.001	4.2 (1.2–10.7)	3.9 (0.9–8.8)	0.01
Kömek et al. (2021) (30)	7		6.0 (3.7–15.1)	205	4.4 (2.5–8.1)	146	<0.001	10.6 (5.8–19.2)	2.1 (1.2–3.9)	<0.001
Chen et al. (2020) (32)	11 (axial skeleton)		9.15 (1.61–29.96)	11 patients	6.15 (1.09–16.66)	7 patients	1.96			
	6 (hip bone)		10.14 (2.59–15.5)	6 patients	4.38 (1.25–6.80)	5 patients	0.046			
	3 (appendicular skeleton)		6.71 (1.69–8.16)	3 patients	4.39 (1.67–7.82)	3 patients	0.273			
Wu et al. (2021) (12)	NR	66 (osteolytic)	10.6	66	6.1	55	<0.01			
	NR	43 (osteoblastic)	7.7	43	3.7	34	<0.01			
Guo et al. (2020) (14)	5	43	6.72 (1.77–27.08)	43	2.83 (0.81–14.31)	33	0.039			
Pang et al. (2020) (28)	5		4.3 (2.3–10.7)	67	2.2 (0.5–6.7)	55	<0.001			
Lan et al. (2022) (29)	18	147	12.88 ± 8.61		7.71 ± 4.76		0.02			

## Results and discussion

Presently, the study on the diagnostic effectiveness of benign and malignant lesions of [^68^Ga]Ga-DOTA-FAPI-04 has been performed across different countries. In several studies, [^68^Ga]Ga-DOTA-FAPI-04 was proven to provide better sensitivity and specific results. However, most of them are small sample tests and are mostly retrospective. Only a few systemic reviews of [^68^Ga]Ga-DOTA-FAPI-04 PET have been performed. When compared with the previous system summary about [^68^Ga]Ga-DOTA-FAPI-04, our systematic review was mainly focused on the head-to-head diagnosis of [^18^F]FDG PET/CT and [^68^Ga]Ga-DOTA-FAPI-04 PET/CT. We have rated the diagnostic efficiency of [^68^Ga]Ga-DOTA-FAPI-04 for tumor bone metastases from the present retrospective studies.

### Analysis of the diagnostic efficiency of the two imaging agents in different primary tumors

The eight studies include different types of malignant physical tumors, such as nasopharyngeal cancer, gastrointestinal malignant tumors, liver cancer, lung cancer, thyroid cancer, and breast cancer. As these studies did not show true positive (TP), false positive (FP), true negative (TN), and false negative (FN) in detail, we did not analyze the diagnostic sensitivity and specificity of the two images in different tumors (12, 14, 27, 29–32). Some of the included studies were classified as the first diagnosis and recurrence type, and some did not fall into any group. However, in all studies, the extracted data showed that the diagnostic sensitivity of [^68^Ga]Ga-DOTA-FAPI-04 PET/CT was better than that of [^18^F]FDG PET/CT for the diagnosis of malignant tumors in patients with a primary diagnosis or recurrence stages. The specificity of [^18^F]FDG was better than that of [^68^Ga]Ga-DOTA-FAPI-04. When compared with [^18^F]FDG in the primary lesion, [^68^Ga]Ga-DOTA-FAPI-04 showed a higher intake, which better displayed the outline of the lesion. [^68^Ga]Ga-DOTA-FAPI-04 showed better tumor-background contrast, which facilitated the easy detection of hidden lesions. Non-specific fibrosis induced by inflammation can also cause the positive intake of [^68^Ga]Ga-DOTA-FAPI-04. Therefore, benign inflammation reactions can be the main factor leading to the [^68^Ga]Ga-DOTA-FAPI-04 PET/CT false-positive results, and it is also the main reason for the specificity of [^68^Ga]Ga-DOTA-FAPI-04 not being as good as that of [^18^F]FDG.

### Analysis of the diagnostic effectiveness of the two imaging agents in different malignant tumor bone metastases

**Figure 4:**
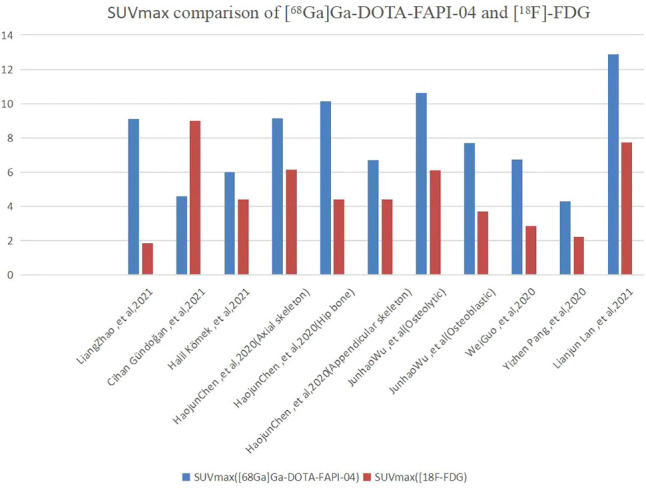
SUVmax comparison of [^68^Ga]Ga-DOTA-FAPI-04 and [^18^F]FDG.

**Figure 5:**
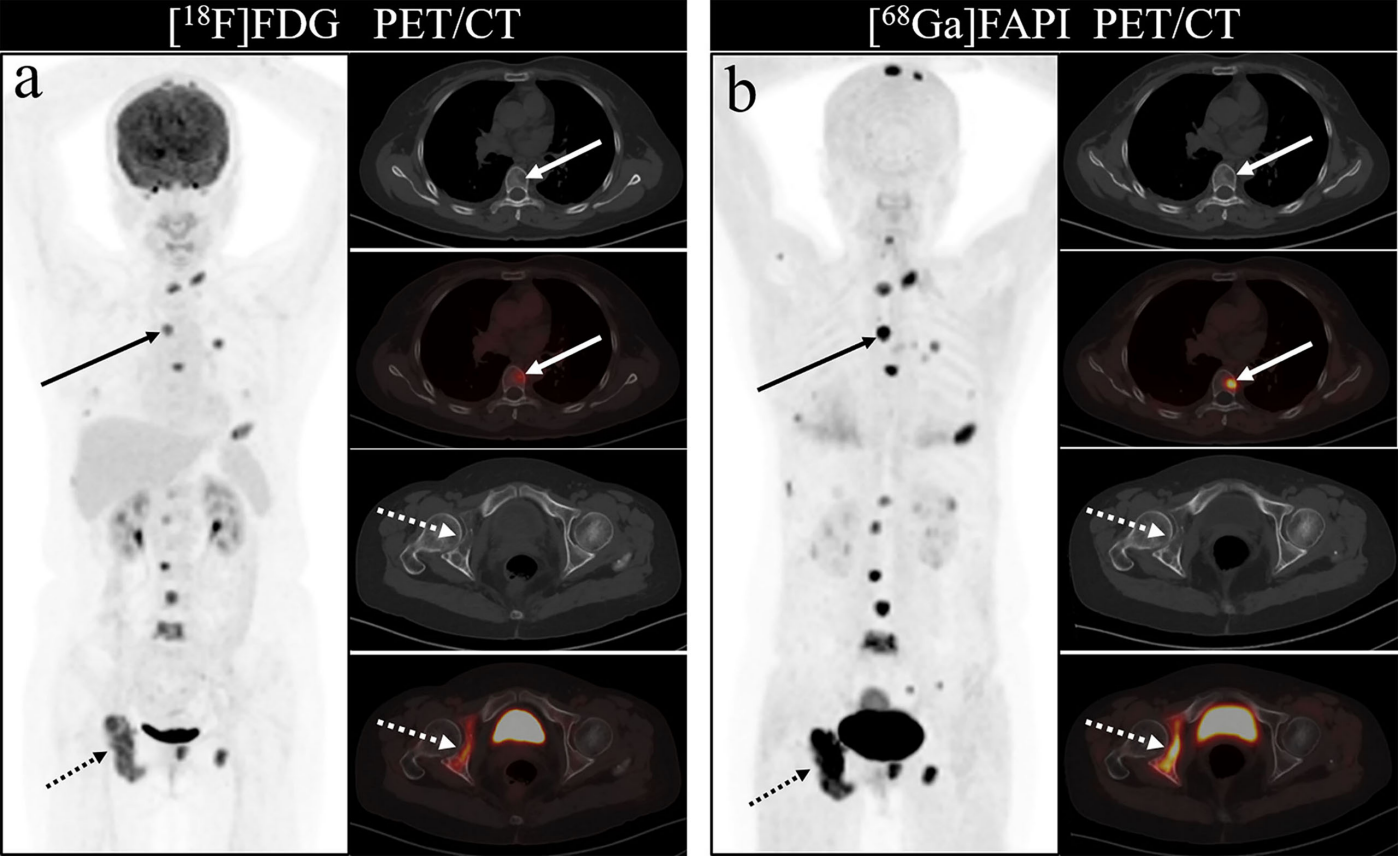
One of the patients’ [^18^F]FDG **(A)** and [^68^Ga]Ga-DOTA-FAPI-04 PET/CT **(B)** images. The figure is dapted from Wu et al. This research was originally published in Frontiers in Oncology.

As shown in **Figure 4**, all eight articles included in this review reported the intake of [^68^Ga]Ga-DOTA-FAPI-04 and [^18^F]FDG in bone metastatic tumors. A total of six reports demonstrated that the maximum standardized uptake value (SUVmax) in the metastasis of [^68^Ga]Ga-DOTA-FAPI-04 was higher than that of [^18^F]FDG, and the difference was statistically significant (*p* ≤ 0.05) (12, 14, 27–31). **Figure 5** offers an overview of the typical performance of [^68^Ga]Ga-DOTA-FAPI-04 and [^18^F]FDG PET/CT in bone lesion diagnosis. **Figure 5** was adapted from Wu et al.; this research was originally published in *Frontiers in Oncology*. Chen et al. reported that the SUVmax in 11 middle-shaft bones and three limbs of [^68^Ga]Ga-DOTA-FAPI-04 was higher than those of [^18^F]FDG; however, the difference was not statistically significant (32). An article by Gündoğan et al. concluded that the SUVmax of [^18^F]FDG in the bone metastases was higher than that of [^68^Ga]Ga-DOTA-FAPI-04 because the metabolic activity of bone metastases was associated with the pathological type of the primary tumor and the metabolic activity of primary tumors. Gündoğan et al. reported gastrointestinal adenocarcinoma as the primary tumor. Therefore, the diagnostic value of [^68^Ga]Ga-DOTA-FAPI-04 PET/CT and [^18^F]FDG PET/CT in different tumor subtypes should be studied (31). Only the studies by Gündoğan et al. and Kömek et al. compared the tumor-background contrast of the two images in the bone metastatic lesion. However, it was concluded that [^68^Ga]Ga-DOTA-FAPI-04 had a higher level of comparison, and *p* ≤ 0.05 was considered to indicate statistical significance (30, 31). Moreover, [^68^Ga]Ga-DOTA-FAPI-04 displayed a better detection performance for bone metastases and detected more bone metastases than [^18^F]FDG. Only one article by Wu et al. studied the intake of the two imaging agents in detail in different pathological types of bone metastases (12). Among them, [^68^Ga]Ga-DOTA-FAPI-04 detected bone metastases in 30 individuals and only 26 were detected in [^18^F]FDG (100% [30/30] *vs*. 86.7% [26/30], *p* = 0.125). Among the 119 bone lesions, 109 were bone metastases. The true-positive value of [^68^Ga]Ga-DOTA-FAPI-04 was 109, and the false-positive value was 10. The true-positive value of [^18^F]FDG was 89, and the false-positive value was 5. Moreover, whether [^68^Ga]Ga-DOTA-FAPI-04 or [^18^F]FDG, SUVmax in the osteolytic lesion was greater than that in a bone lesion. The reason for this result is not clear; therefore, further research is required.

### Analysis of the intake of [^68^Ga]Ga-DOTA-FAPI-04 and [^18^F]FDG in benign diseases

In all eight articles, the intake of the two imaging agents in benign lesions was mentioned. In these studies, [^68^Ga]Ga-DOTA-FAPI-04 had a higher SUVmax in bone benign lesions, which also led to a higher false-positive rate in the diagnosis of bone metastases by [^68^Ga]Ga-DOTA-FAPI-04. This included bone marrow fibrosis, degenerative osteophytes, osteoarthritis, and fractures, which were observed with high [^68^Ga]Ga-DOTA-FAPI-04 intake. Gündohan et al. and Pang et al. reported that the fibrosis caused by some radioactive therapy and surgery had a high intake of [^68^Ga]Ga-DOTA-FAPI-04 (28, 31). Zhao et al., Kömek et al., and Lan et al. reported that in some internal organ fibrosis, such as liver and kidney fibrosis, pancreatitis, and breast hyperplasia, [^68^Ga]Ga-DOTA-FAPI-04 had a high intake (27, 29, 30). Moreover, [^68^Ga]Ga-DOTA-FAPI-04 and [^18^F]FDG had a high intake in some inflammatory granulomas, lung infections, tuberculosis, and inflammatory lymph nodes.

### Analysis of the heterogeneity

Several differences were observed among the eight articles. One article studied patients with nasopharyngeal carcinoma, one studied patients with gastric adenocarcinoma, and another one studied patients with breast cancer; two articles studied patients with various types of cancer; one studied patients with lung cancer, thyroid cancer, and liver cancer; one studied patients with liver cancer; one studied patients with gastric, duodenal, and colorectal cancers; and another one did not mention the median of the patient’s age. However, all articles gave the scope of the patient’s age. All articles were inconsistent in terms of the gender and age of patients. The activity standards of [^68^Ga]Ga-DOTA-FAPI-04 were not uniform. One article reported the total injection activity range. Some studies reported the scope of weight-based injection activity, and one article did not mention relevant information. The time from the radiopharmaceutical injection to PET image acquisition was different. One article was ≥40 min, five articles were ≥60 min, and two articles did not mention the time. The scan interval times were different. One article’s scan interval was 3 days, one article’s interval was 1–6 days, another article’s interval was 1–14 days, and five articles’ interval was <1 week.

The results indicated that the diagnostic sensitivity of [^68^Ga]Ga-DOTA-FAPI-04 was better than that of [^18^F]FDG PET/CT. [^68^Ga]Ga-DOTA-FAPI-04 PET/CT was cheaper than [^18^F]FDG PET/CT. Patients fasted for >6 h before [^18^F]FDG PET/CT, whereas no specific preparation was required for [^68^Ga]Ga-DOTA-FAPI-04 PET/CT (27).

This study also has certain limitations. The sample size of the trial was small. In addition, these trials were heterogeneous in terms of the pathological type of the primary tumor, the radiopharmaceutical injected activity, time from the radiopharmaceutical injection to PET image acquisition, and PET image analysis methods for qualitative (visual) analysis. The heterogeneity was significant. The results of the systematic review do not completely represent the results of [^68^Ga]Ga-DOTA-FAPI-04 PET/CT and [^18^F]FDG PET/CT in the diagnostic value of malignant tumor bone metastasis. Therefore, a greater number of studies on bone metastasis are required in the future.

## Conclusion

In this retrospective study, we evaluated the diagnostic effectiveness of [^68^Ga]Ga-DOTA-FAPI-04 in the diagnosis of tumor bone metastasis. The results showed that the diagnostic sensitivity of [^68^Ga]Ga-DOTA-FAPI-04 may be better than that of [^18^F]FDG PET/CT. [^68^Ga]Ga-DOTA-FAPI-04 FAPI showed better detection performance for bone metastases; however, its false-positive rate was higher for the diagnosis of bone metastases. Therefore, a high-quality, multicentral, and multigroup random control study is required in the future to completely determine the value of [^18^F]FDG to detect bone metastasis in patients with different cancer types.

## Data availability statement

The original contributions presented in the study are included in the article/supplementary material. Further inquiries can be directed to the corresponding author.

## Author contributions

LL, JM, and CZ contributed to conception and design of the study. JM organized the database. JM and XH performed the statistical analysis. JM wrote the first draft of the manuscript. LL, XH, and SY wrote sections of the manuscript. All authors contributed to the article and approved the submitted version.

## Conflict of interest

The authors declare that the research was conducted in the absence of any commercial or financial relationships that could be construed as a potential conflict of interest.

## Publisher’s note

All claims expressed in this article are solely those of the authors and do not necessarily represent those of their affiliated organizations, or those of the publisher, the editors and the reviewers. Any product that may be evaluated in this article, or claim that may be made by its manufacturer, is not guaranteed or endorsed by the publisher.
